# Longitudinal Nutritional and Cardiorenal Changes Among Patients Prescribed a Ketoanalogue-Supplemented Very-Low-Protein Diet in Advanced Nondialysis Chronic Kidney Disease with Chronic Heart Failure: A Retrospective Cohort Study

**DOI:** 10.3390/nu18183069

**Published:** 2026-09-20

**Authors:** Maria-Daniela Tanasescu, Andrei-Mihnea Rosu, Alexandru Minca, Elena-Iuliana Andrieș (Ioniță), Delia Timofte, Lunca Dragos Constantin, Daniel Iulian Voiculescu, Dorin Ionescu

**Affiliations:** 1 Department of Semiology-Emergency University Hospital, Carol Davila University of Medicine and Pharmacy, 022328 Bucharest, Romania; maria.tanasescu@umfcd.ro (M.-D.T.); dorin.ionescu@umfcd.ro (D.I.); 2 Department of Cardiology, Prof. Dr. Agrippa Ionescu Emergency Hospital, 077015 Balotesti, Romania; 3 Department of Pharmacognosy, Phytochemistry and Phytotherapy, Faculty of Pharmacy, Carol Davila University of Medicine and Pharmacy, 020021 Bucharest, Romania; elena.ionita@umfcd.ro; 4 Department of Dialysis, Bucharest Emergency University Hospital, 050098 Bucharest, Romania; delia.timofte@gmail.com; 5 Department of Pharmacology, Clinical Pharmacology and Pharmacotherapy, Faculty of Pharmacy, Carol Davila University of Medicine and Pharmacy, 020021 Bucharest, Romania; dragos.lunca@umfcd.ro; 6 Department of General Surgery No 1—Emergency University Hospital, Carol Davila University of Medicine and Pharmacy, 022328 Bucharest, Romania; daniel.voiculescu@umfcd.ro

**Keywords:** chronic kidney disease, ketoanalogues, very-low-protein diet, dietary adherence, nutritional status, protein–energy wasting, heart failure, cardiorenal syndrome

## Abstract

**Background/Objectives:** Ketoanalogue-supplemented very-low-protein diets may reduce metabolic and mineral burden in advanced chronic kidney disease (CKD), but nutritional changes and adherence remain concerns. This study evaluated nutritional, renal, mineral–bone, hematologic, and cardiovascular changes in patients with advanced nondialysis CKD and chronic heart failure receiving this dietary regimen. **Methods:** This single-center retrospective cohort included 75 adults followed for 181–730 days, with a median follow-up of 243 days (interquartile range [IQR], 212–365 days). Prescribed protein and energy targets were 0.28–0.40 g protein/kg/day and 28–34 kcal/kg/day, respectively, with approximately one ketoanalogue tablet per 5 kg body weight/day and dietitian involvement. Paired analyses and exact-date body-weight sensitivity analyses were performed. Clinician-recorded adherence was summarized descriptively because assessment methods were not standardized. **Results:** The prespecified primary outcome, percentage body-weight change, had a Hodges–Lehmann estimate of −3.99% (95% CI, −4.68 to −3.42; *p* < 0.001). Mean body weight decreased from 75.85 ± 7.98 to 72.65 ± 7.09 kg, with a Hodges–Lehmann estimate of −3.10 kg (95% confidence interval [CI], −3.60 to −2.65; *p* < 0.001). Body mass index (BMI) also declined (estimate, −0.95 kg/m^2^; *p* < 0.001). A body-weight reduction of ≥5% occurred in 22 patients (29.3%); none had a reduction of ≥10%. The decrease remained evident among 62 patients followed for 181–366 days. Serum albumin and total protein increased slightly, while chart-recorded signs suggestive of nutritional deterioration were present in 25 patients (33.3%). Absolute eGFR change was modest (Hodges–Lehmann estimate, −1.50 mL/min/1.73 m^2^; 95% CI, −2.50 to −0.50; *p* = 0.007). Phosphorus, calcium–phosphorus product, intact parathyroid hormone, and alkaline phosphatase decreased, while calcium increased. Blood pressure showed little change, NYHA functional class was unchanged in 61 patients (81.3%), and left ventricular ejection fraction (LVEF) declined slightly. No maintenance dialysis initiation or death was documented before the final assessment. **Conclusions:** Body weight and BMI declined despite small cohort-level increases in serum proteins. Multidimensional nutritional monitoring and interpretation of weight change alongside fluid status are warranted. The absence of a control group, variable follow-up, and nonstandardized adherence assessment preclude causal interpretation.

## 1. Introduction

Advanced chronic kidney disease (CKD) is accompanied by metabolic, nutritional, and cardiovascular complications that frequently overlap. Heart failure (HF) affects approximately 10–30% of patients with CKD, while CKD is present in 30–60% of patients with HF; their coexistence is associated with more frequent hospitalization, faster renal decline, and higher mortality [1]. Hypertension, diabetes, inflammation, oxidative stress, endothelial dysfunction, and volume overload contribute to this cardiorenal interaction [2]. Nutritional vulnerability is also common, as uremic symptoms, metabolic acidosis, gastrointestinal discomfort, inflammation, poor appetite, and dietary restrictions may reduce intake and promote catabolism. Protein–energy wasting (PEW) has been reported in 11–54% of patients with CKD stages 3–5 [3]. Its recognition is particularly difficult in patients with HF because edema and changes in hydration status may obscure tissue loss, while serum albumin is influenced by inflammation and fluid status and does not directly reflect changes in muscle or fat mass [4].

Protein restriction is used to reduce nitrogenous waste production, dietary phosphorus exposure, and the metabolic burden of advanced CKD [5]. Conventional low-protein diets generally provide 0.55–0.80 g/kg/day, whereas very-low-protein diets provide approximately 0.28–0.43 g/kg/day and require supplementation with ketoanalogues or essential amino acids [6]. Ketoanalogues provide precursors for essential amino acid synthesis without an equivalent nitrogen load. Supplemented protein restriction has been associated with slower kidney-function decline, lower phosphorus and urea concentrations, and preservation of several nutritional indices [6]. Reduced risks of rapid eGFR decline and dialysis initiation have also been reported [7], together with lower concentrations of selected uremic toxins, higher bicarbonate levels, and improved endothelial function [8]. These potential benefits depend on adequate energy intake and clinical supervision, as insufficient caloric intake may increase protein catabolism and the risk of nutritional deterioration [9].

Implementation remains demanding because the regimen requires sustained changes in food selection, meal preparation, protein intake, energy provision, and daily tablet use. Dietary intake may also be affected by appetite limitation, gastrointestinal symptoms, functional impairment, treatment burden, food costs, social eating, and family support [10,11]. Individualized counseling and practical dietary guidance may improve acceptance, while structured education can facilitate protein restriction without reducing patient satisfaction [11,12]. Nevertheless, adherence is difficult to measure consistently in routine care. Food diaries, dietary recalls, self-monitoring applications, and clinician estimates may capture different aspects of dietary behavior, and difficulty following the regimen may occur alongside worsening appetite, frailty, or intercurrent illness. Observational associations between recorded adherence and nutritional findings therefore require cautious interpretation because the temporal direction of the association may be uncertain. Nutritional monitoring should include body-weight trajectory, reported intake, appetite, biochemical markers, muscle-related findings, functional status, and fluid balance rather than relying on a single measure.

Evidence from routine clinical practice remains limited, particularly in patients with advanced CKD and concomitant HF, in whom changes in body weight may reflect both nutritional and volume-status changes [13,14]. The present study described longitudinal anthropometric, biochemical, renal, CKD–mineral and bone disorder, hematologic, and cardiovascular changes in adults with advanced nondialysis CKD and chronic HF receiving a ketoanalogue-supplemented very-low-protein diet. Exploratory analyses examined factors associated with percentage body-weight change and annualized eGFR change. Clinician-recorded adherence was summarized descriptively and examined only in exploratory analyses because its assessment was not standardized across the cohort.

## 2. Materials and Methods

### 2.1. Study Design, Setting, and Ethical Approval

This single-center retrospective observational cohort study was conducted in the Nephrology Department of the Emergency University Hospital Bucharest, Romania. The study included adult patients with CKD who were evaluated and followed between April 2021 and June 2026. Data were obtained from routinely collected medical records, including demographic and clinical information, laboratory measurements, nutritional assessments, echocardiographic examinations, cardiovascular diagnoses, and pharmacological treatment. Baseline and follow-up variables were extracted from the available hospital and outpatient records and entered into a standardized anonymized database. Information from nutritional, renal, laboratory, medication, and cardiovascular records was linked using coded patient identifiers. Ancillary source files were used to verify or supplement information for patients in the source population but were not considered separate recruitment populations. Patient names and other direct identifiers were removed before statistical analysis, and each participant was assigned a coded study identification number. The study was conducted in accordance with the principles of the Declaration of Helsinki and reported according to the Strengthening the Reporting of Observational Studies in Epidemiology recommendations [15]. Ethical approval was obtained from the Ethics Committee of the Emergency University Hospital Bucharest (approval number 49715/approval date 30 June 2026). Due to the retrospective design and use of anonymized data, the requirement for individual informed consent was waived.

### 2.2. Patient Selection

The study cohort was constructed by linking the renal/nutritional treatment database with the cardiovascular database using coded patient identifiers. During the predefined study period, 156 unique patients who had been prescribed the ketoanalogue-supplemented very-low-protein dietary protocol were identified across the available source records. The final linked cohort comprised 75 patients who were represented in both databases and had been prescribed the ketoanalogue-supplemented very-low-protein dietary protocol. Thus, concomitant chronic heart failure was a cohort-defining characteristic of the linked dataset rather than an incidental finding after eligibility assessment. Patients were not selected according to HF phenotype, NYHA functional class, or the subsequent direction or magnitude of nutritional, renal, or cardiovascular changes.

Eligibility additionally required age ≥18 years, established nondialysis CKD, at least six months of follow-up, and baseline and final measurements of body weight, BMI, serum albumin, total protein, and eGFR. All 75 patients in the final linked cohort fulfilled these requirements, and none was excluded after cohort formation. Baseline was defined as the first eligible assessment at initiation or confirmation of the supplemented dietary protocol, and final follow-up as the last eligible assessment recorded no later than June 2026.

**Figure 1:**
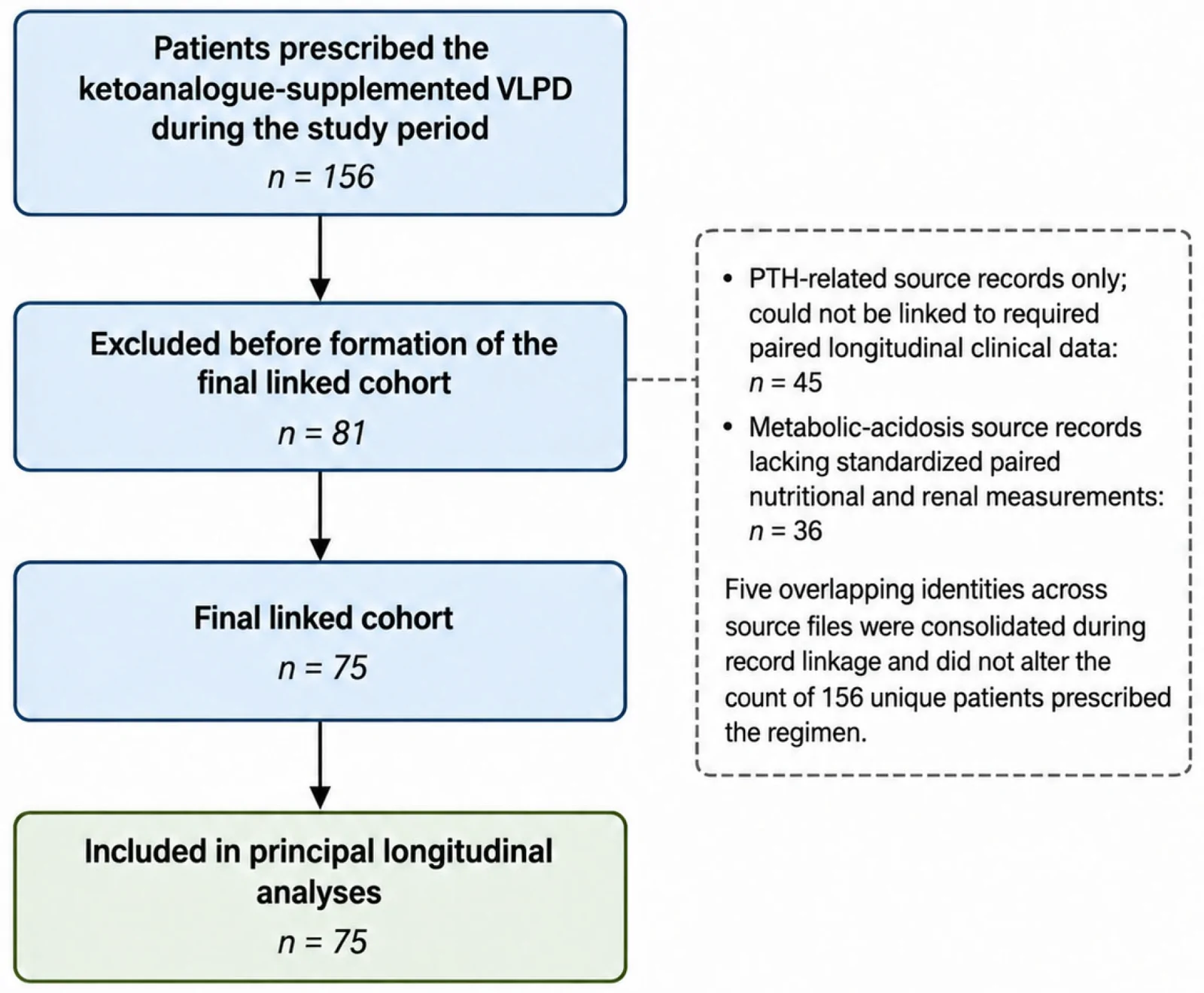
Patient flow diagram.

Before formation of the final linked cohort, 81 of the 156 patients prescribed the regimen were excluded because the available records did not permit inclusion in the longitudinal analysis. Forty-five patients were represented only in the PTH-related source records and could not be linked to the required paired longitudinal clinical data, while 36 patients were represented in the metabolic-acidosis source records but lacked the standardized paired nutritional and renal measurements required for the present analysis. Five overlapping identities across the source files were consolidated during record linkage and did not alter the count of 156 unique patients prescribed the regimen. All 75 patients had the paired nutritional and renal measurements required for the principal analyses (Figure 1).

### 2.3. Dietary Intervention and Clinician-Recorded Adherence

All patients received an individualized CKD-specific very-low-protein diet with prescribed energy target of 0.28–0.40 g/kg actual body weight/day and prescribed an energy target of 28–34 kcal/kg/day. These values represented prescribed targets rather than systematically measured nutrient intake. Ketoanalogues were prescribed at approximately one tablet per 5 kg actual body weight/day, rounded to the nearest whole tablet; recorded prescriptions ranged from 11 to 19 tablets/day. Dietary counseling was provided by the nephrology team with dietitian involvement, with reassessment according to clinical need.

Adherence was available as a clinician-recorded numeric percentage reflecting the overall dietary and ketoanalogue regimen. When documented, assessment was based on a self-monitoring application, food diary, or dietary recall; the assessment method was unspecified in 45 of 75 patients. Adherence was therefore considered a nonvalidated routine-care estimate and categorized as high (≥90%), intermediate (70–89%), or low (<70%). Because assessment methods and timing were not standardized, adherence categories were used only for descriptive supplementary summaries and were not treated as comparative exposure groups; incorporation bias and reverse causation could not be excluded.

Dietitian records were also reviewed for appetite, dietary tolerance, muscle-related findings, and supportive interventions, including intensified counseling or consideration of oral nutritional supplementation.

### 2.4. Data Sources and Quality Control

Clinical, nutritional, laboratory, echocardiographic, and medication records were linked using anonymized study identifiers. Follow-up duration was calculated from verified baseline and final assessment dates and expressed in days; years were calculated using 365.25 days for time-standardized analyses. Eligibility did not require complete paired data for every secondary longitudinal variable; completeness was assessed after record linkage, and variables retained in the paired longitudinal analyses had both baseline and follow-up values available for all 75 patients.

Discordant or repeated entries were checked against the dated source record, with the original clinical source given priority over secondary compiled files. No predefined maximum time window was applied for laboratory or echocardiographic measurements relative to the baseline or final assessment. When measurements were obtained on different dates, the value closest to the corresponding assessment date was retained. To characterize temporal alignment, the absolute interval between each retained measurement and the corresponding assessment date was calculated. Baseline and follow-up laboratory measurements were obtained a median of 2 (1–4) and 3 (1–4) days from the corresponding assessment dates, respectively (ranges, 0–5 and 0–5 days). The corresponding intervals for baseline and follow-up echocardiography were 4 (2.5–8) and 6 (3–8) days, respectively (ranges, 0–10 and 0–10 days). Implausible values were re-examined before database finalization. Derived variables were recalculated from source data and checked for internal consistency. Missing values were not imputed, and analyses were performed using available cases.

Operational definitions, units, coding rules, and data sources are presented in Supplementary Table S1. The numbers of available baseline, follow-up, and paired observations for all variables included in longitudinal analyses are reported in Supplementary Table S2.

### 2.5. Nutritional Variables and Safety Outcomes

Nutritional assessment included body weight, BMI, serum albumin, total protein, appetite-related observations, and available muscle-related findings. Percentage body-weight change was calculated relative to baseline. Reductions of ≥5% and ≥10% were evaluated descriptively as anthropometric outcomes and were not considered diagnostic of malnutrition or PEW.

Muscle-related findings were classified from routine records as absent, a slight reduction in mid-arm muscle circumference without documented functional impairment, or reduced mid-arm muscle circumference accompanied by lower handgrip strength. Because standardized nutritional assessment tools and complete PEW diagnostic criteria were not applied consistently, chart-recorded findings were described as signs suggestive of nutritional deterioration or PEW rather than confirmed PEW. Body-weight and serum-protein changes were interpreted cautiously because hydration and congestion could influence both measures.

### 2.6. Renal, Mineral–Bone, Hematologic, and Cardiovascular Variables

Renal variables included serum creatinine, urea, and eGFR at baseline and follow-up. CKD G categories were defined according to KDIGO criteria. Absolute eGFR change was calculated as the final minus baseline value, while annualized eGFR change was calculated using each patient’s verified follow-up duration and treated as a time-standardized sensitivity estimate rather than an individual renal slope. CKD G-category transitions were classified descriptively as less advanced, stable, or more advanced.

CKD–MBD variables included calcium, phosphorus, calcium–phosphorus product (Ca × P), iPTH, and ALKP. Hematologic assessment included hemoglobin, anemia grade, and documented iron deficiency.

Cardiovascular variables included HF phenotype, NYHA functional class, LVEF, NT-proBNP, AF, CAD, blood pressure, valvular disease, PASP, orthostatic hypotension, hypotension episodes, and loop-diuretic treatment. HF phenotype was classified as HFrEF (LVEF ≤ 40%), HFmrEF (41–49%), or HFpEF (≥50%) [16,17,18]. Because serial NT-proBNP values were not consistently available, NT-proBNP was treated as a baseline marker. HF decompensation, hospitalization, edema, and congestion were reviewed when documented but were not assessed systematically at standardized intervals. Loop-diuretic dose changes and standardized serial measures of volume status, dry weight, bioimpedance, or body composition were unavailable.

Concomitant treatments were reviewed from the available clinical and medication records. Loop-diuretic exposure was recorded, but dose changes were not captured consistently. Systematic baseline-to-follow-up data on phosphate binders, vitamin D compounds, calcimimetics, calcium supplementation, bicarbonate therapy, renin–angiotensin system inhibitors, SGLT2 inhibitors, mineralocorticoid receptor antagonists, and other treatment modifications were not available in a sufficiently standardized form for longitudinal analysis.

### 2.7. Study Outcomes

The primary outcome was percentage body-weight change from baseline to final follow-up. Because observation time varied, annualized percentage body-weight change was evaluated as a sensitivity outcome, and the body-weight analysis was repeated in patients followed for 181–366 days. Percentage body-weight change was selected as the primary outcome because body weight was routinely and consistently documented at both assessment points for all included patients, and percentage change accounts for differences in baseline body size. It was interpreted as an anthropometric outcome rather than as a direct measure of nutritional tissue loss, particularly because fluid status may influence body weight in patients with HF. A structured analysis plan identifying percentage body-weight change as the primary outcome was established before the final statistical analyses. Because this was a retrospective study of an existing clinical cohort, the sample size was determined by the number of eligible patients available during the predefined study period; no a priori power-based sample-size calculation was performed.

Key secondary outcomes were changes in BMI, serum albumin, and total protein, body-weight reduction ≥5%, and absolute eGFR change from baseline to final follow-up. Annualized eGFR change was evaluated as a time-standardized sensitivity estimate rather than as an individual renal slope. Body-weight reduction ≥10%, chart-recorded appetite and muscle-related findings, CKD G-category transitions, creatinine, urea, CKD–MBD variables, hemoglobin, LVEF, NYHA functional class, and blood pressure were considered exploratory outcomes.

Clinician-recorded adherence categories were used only to provide descriptive supplementary summaries and were not treated as comparative exposure groups. Exploratory outcomes were interpreted primarily according to the magnitude and precision of the observed estimates rather than statistical significance alone.

### 2.8. Statistical Analysis

Continuous variables were summarized as mean ± standard deviation or median [interquartile range], according to distribution, and categorical variables as number (percentage). Baseline-to-follow-up continuous variables were compared using paired-samples *t*-tests or Wilcoxon signed-rank tests. Changes were reported primarily as effect estimates with 95% confidence intervals. For normally distributed paired differences, mean differences with 95% confidence intervals were reported; for non-normally distributed paired differences, Hodges–Lehmann paired-location estimates with percentile-bootstrap 95% confidence intervals based on 10,000 resamples were used. Because CKD G-category and NYHA-class transition tables contained sparse cells, these changes were summarized descriptively as improved/less advanced, stable, or worsened/more advanced, without formal inferential testing.

The association between follow-up duration and percentage body-weight change was assessed using Spearman’s rank correlation. Annualized body-weight and eGFR changes were compared with zero using one-sample Wilcoxon signed-rank tests. The body-weight analysis restricted to 181–366 days used the same paired approach as the complete cohort. Annualized estimates were treated as sensitivity analyses rather than measured longitudinal slopes.

Analyses were interpreted according to the outcome hierarchy defined in the preceding section. Statistical inference centered on the prespecified primary outcome, percentage body-weight change. Key secondary outcomes were interpreted with emphasis on effect estimates and 95% confidence intervals, whereas the remaining outcomes were considered exploratory. No adjustment for multiple comparisons was applied; consequently, *p*-values for secondary, sensitivity, and exploratory analyses were interpreted descriptively and were not used alone to determine the strength of evidence. Adherence-stratified outcomes were presented only as descriptive supplementary data and were not subjected to formal between-group hypothesis testing because clinician-recorded adherence was nonstandardized and follow-up duration differed substantially across categories.

## 3. Results

### 3.1. Cohort Selection and Data Completeness

The final analytical cohort comprised 75 patients. All patients had the paired nutritional and renal measurements required for the principal longitudinal analyses. Completeness of the additional variables retained in paired longitudinal analyses was confirmed after record linkage and was not an additional eligibility criterion. Follow-up ranged from 181 to 730 days, with a median duration of 243 (212–365) days, corresponding approximately to 8 (7–12) months. Sixty-two patients (82.7%) had follow-up intervals of 181–366 days and were included in the restricted-duration body-weight sensitivity analysis. No maintenance dialysis initiation or death was documented before the final follow-up assessment.

### 3.2. Baseline Clinical and Nutritional Characteristics

The cohort comprised 75 patients, including 31 with high adherence, 30 with intermediate adherence, and 14 with low adherence. Median age was 68 (60–74) years, 45 patients (60.0%) were men, and 28 (37.3%) had diabetes mellitus. Most patients had CKD G4 at baseline (64.0%), with a mean eGFR of 22.2 ± 8.1 mL/min/1.73 m^2^. Mean body weight was 75.85 ± 7.98 kg, mean BMI was 26.9 ± 3.8 kg/m^2^, median serum albumin was 3.72 (3.58–3.83) g/dL, and median total protein was 6.80 (6.50–7.00) g/dL.

All patients had chronic HF. HFmrEF was the most frequent phenotype (49.3%), followed by HFpEF (37.3%) and HFrEF (13.3%). Most patients were in NYHA class II (73.3%), and mean LVEF was 48.0 ± 6.5%. AF and CAD were present in 28.0% and 48.0% of patients, respectively.

Baseline characteristics according to clinician-recorded adherence category are presented descriptively in Table 1. Descriptive variation across the categories was observed in several baseline characteristics, including serum albumin, total protein, HF phenotype, and NYHA functional class. Because adherence assessment was nonstandardized and the groups were small and unequal, no formal between-group hypothesis testing was performed, and these descriptive differences were not interpreted as evidence of statistically distinct adherence groups.

### 3.3. Dietary Prescription, Ketoanalogue Exposure, and Adherence

Median prescribed protein and energy intakes were 0.30 [0.30–0.35] g/kg/day and 31 [19,20,21,22,23] kcal/kg/day, respectively. The median ketoanalogue dose was 15 [13.5–16] tablets/day, corresponding to 0.198 [0.196–0.203] tablets/kg/day, and the median treatment duration was 8 (7–12) months. The absolute and weight-adjusted doses according to CKD G category are shown descriptively in Table 2.

The median clinician-recorded adherence estimate was 88% (73.5–92.5). High, intermediate, and low adherence were documented in 31 (41.3%), 30 (40.0%), and 14 (18.7%) patients, respectively. The assessment method was specified in 30 patients: 12 used self-monitoring applications, 12 food diaries, and six dietary recalls. In the remaining 45 patients, a numeric adherence estimate was recorded without documentation of the assessment method. The adherence percentages and categories were therefore treated as descriptive chart-derived variables rather than validated measures of dietary intake or treatment fidelity.

Enhanced dietitian intervention was documented in 25 patients (33.3%). Appetite-related findings were recorded in 32 (42.7%), including reduced or fluctuating appetite in 20 and improvement in 12. Intensified dietary counseling was documented in 15 patients (20.0%), and oral nutritional supplementation was considered in 10 (13.3%). These supportive measures were summarized descriptively, without formal comparisons across CKD G categories. Nutritional prescription, ketoanalogue exposure, and adherence are summarized in Table 2.

### 3.4. Anthropometric, Biochemical, and Chart-Recorded Nutritional Changes

Body weight decreased during follow-up. In the complete cohort, mean body weight declined from 75.85 ± 7.98 to 72.65 ± 7.09 kg. The median individual change was −3.00 (−4.55 to −1.50) kg, with a Hodges–Lehmann estimate of −3.10 kg (95% CI, −3.60 to −2.65; *p* < 0.001). Median percentage body-weight change was −3.87% (−6.07 to −2.11). For the prespecified primary outcome, the Hodges–Lehmann estimate of percentage body-weight change was −3.99% (95% CI, −4.68 to −3.42; *p* < 0.001). At the cohort level, serum albumin and total protein increased slightly. The mean paired increase in albumin was 0.075 g/dL (95% CI, 0.048–0.102; *p* < 0.001), while the Hodges–Lehmann estimate for total protein change was 0.05 g/dL (95% CI, 0.05–0.10; *p* < 0.001). Individual trajectories varied, with increases, stable values, and decreases observed for both biochemical measures. The magnitude of the cohort-level changes was small and was not interpreted independently as evidence of improved or preserved nutritional status.

A body-weight reduction of at least 5% occurred in 22 patients (29.3%) in the complete cohort. No patient experienced a reduction of at least 10% of baseline body weight. Documented signs suggestive of PEW were present in 25 patients (33.3%), including mild findings in 15 (20.0%) and mild–moderate findings in 10 (13.3%). Reduced or fluctuating appetite was documented in 20 patients (26.7%), while appetite improvement was recorded in 12 (16.0%). Muscle-related findings were present in 19 patients (25.3%): 9 had a slight reduction in mid-arm muscle circumference without documented functional impairment, and 10 had reduced mid-arm muscle circumference accompanied by lower handgrip strength. These findings reflected routine chart documentation and were not interpreted as confirmed PEW.

**Figure 2:**
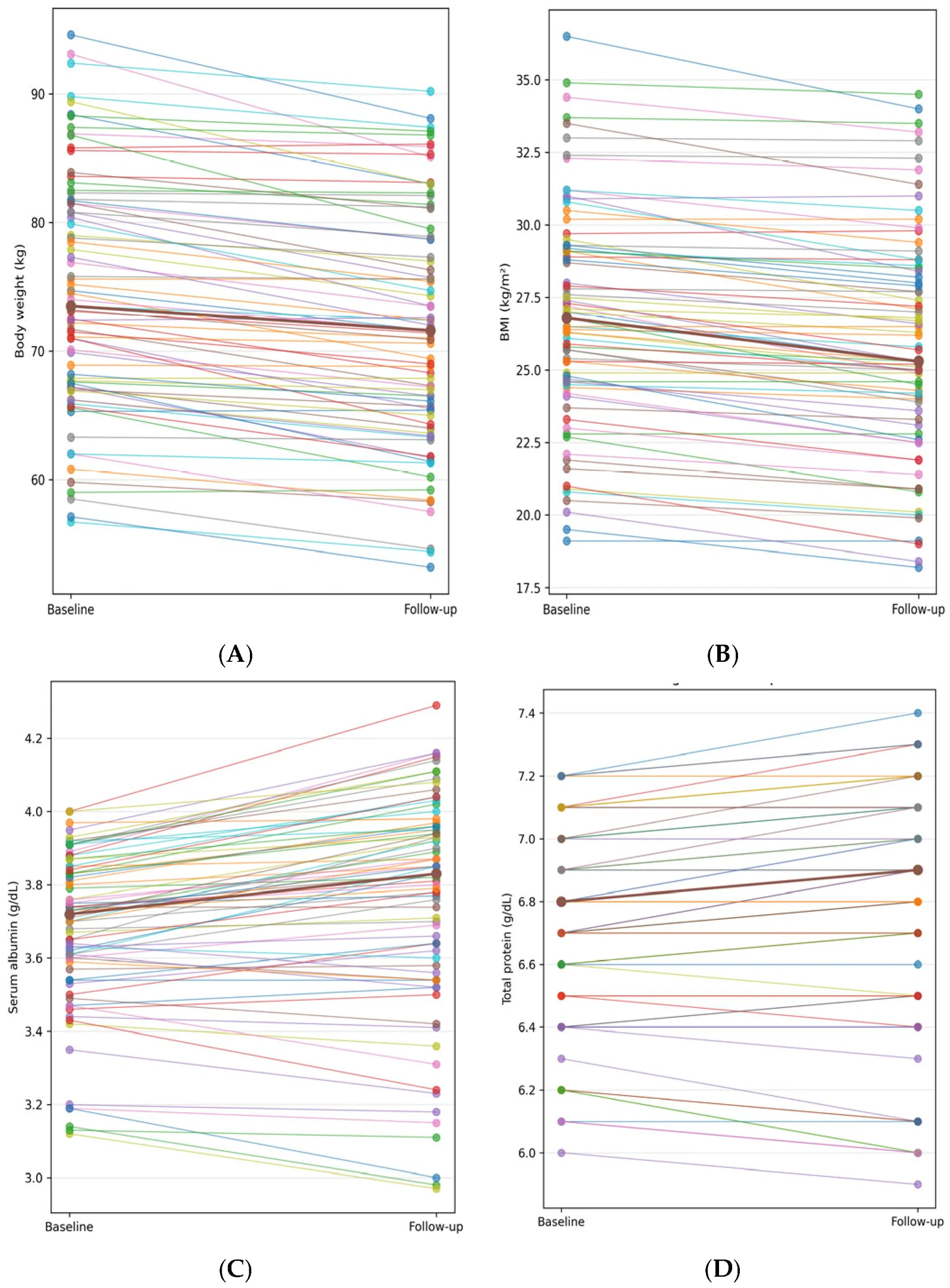
Individual paired anthropometric and biochemical trajectories during follow-up. Paired baseline and follow-up values are shown for (**A**) body weight, (**B**) body mass index, (**C**) serum albumin, and (**D**) total protein in the 75 included patients. Each line connects measurements obtained from the same participant. The trajectories show individual variability and do not distinguish changes attributable to fluid volume from changes in body tissue. The larger superimposed markers and connecting lines indicate the cohort medians at baseline and follow-up.

Because standardized serial measures of congestion, body composition, or dry weight were unavailable, the observed reductions in body weight and BMI could not be divided into changes in fluid volume, adipose tissue, and muscle mass. Paired nutritional changes are presented in Table 3, and individual trajectories are shown in Figure 2.

### 3.5. Time-Adjusted Body-Weight Analyses

Exact follow-up duration was calculated from the baseline and final assessment dates. Follow-up ranged from 181 to 730 days, with a median of 243 (212–365) days. Longer follow-up was associated with a greater cumulative percentage body-weight reduction (Spearman’s *ρ* = −0.592, *p* < 0.001), indicating that observation time influenced the magnitude of the baseline-to-follow-up change.

In the complete cohort, median annualized percentage body-weight change was −4.99%/year (−7.13 to −3.09). The Hodges–Lehmann estimate was −5.08%/year (95% CI, −5.71 to −4.47; *p* < 0.001 versus zero). Because annualization assumes an approximately linear trajectory between the two assessment points, this result was considered a supportive sensitivity finding rather than a replacement for the observed baseline-to-follow-up change.

### 3.6. Body-Weight Findings According to Follow-Up Duration

The prespecified restricted-duration sensitivity analysis included the 62 patients followed for 181–366 days, providing a more homogeneous observation interval. In this subgroup, mean body weight decreased from 75.57 ± 8.06 to 72.95 ± 7.20 kg. The median paired change was −2.85 (−3.50 to −1.30) kg, with a Hodges–Lehmann estimate of −2.55 kg (95% CI, −3.00 to −2.15; *p* < 0.001). Median percentage body-weight change was −3.60% (−4.24 to −1.83), and 10 of 62 patients (16.1%) had a reduction of at least 5%; none had a reduction of at least 10%.

Body-weight findings were also summarized separately for the 13 patients followed for >366 days (Table 4). Mean body weight decreased from 77.19 ± 7.75 to 71.22 ± 6.62 kg in this subgroup, compared with 75.57 ± 8.06 to 72.95 ± 7.20 kg among patients followed for 181–366 days. A body-weight reduction of at least 5% occurred in 12 of 13 patients (92.3%) with >366 days of follow-up and in 10 of 62 patients (16.1%) followed for 181–366 days. These duration-stratified results are presented descriptively without formal between-group testing. The larger cumulative reduction observed in the longer-follow-up subgroup should not be interpreted as a greater rate of body-weight change because observation periods differed substantially and only baseline and final body-weight measurements were consistently available. In addition, body-weight changes in patients with heart failure may reflect changes in fluid status as well as changes in body tissue.

### 3.7. Renal, Mineral–Bone, Hematologic, and Cardiovascular Changes

Renal function declined modestly during follow-up. Median eGFR decreased from 22.0 [16.0–26.5] to 21.0 [14.5–25.0] mL/min/1.73 m^2^, with a Hodges–Lehmann estimate of −1.50 mL/min/1.73 m^2^ (95% CI, −2.50 to −0.50; *p* = 0.007). Median annualized eGFR change was −1.71 [−6.00 to 1.66] mL/min/1.73 m^2^/year, with a Hodges–Lehmann estimate of −1.96 mL/min/1.73 m^2^/year (95% CI, −3.25 to −0.64; *p* = 0.007). Because this value was derived from only the baseline and final measurements, it represents a mathematical time-standardized approximation rather than a measured renal slope. Serum creatinine increased modestly (*p* = 0.004), whereas serum urea did not change significantly (*p* = 0.588).

CKD G category remained unchanged in 58 patients (77.3%). Seven patients (9.3%) moved to a less advanced category, and 10 (13.3%) moved to a more advanced category. These transitions were based on the two available assessment points and were interpreted descriptively.

Calcium increased from 8.44 ± 0.77 to 8.85 ± 0.60 mg/dL, while phosphorus, the Ca × P, iPTH, and ALKP decreased significantly (all *p* < 0.001). Hemoglobin remained stable, with a mean paired change of 0.08 g/dL (95% CI, −0.02 to 0.18; *p* = 0.097).

**Figure 3:**
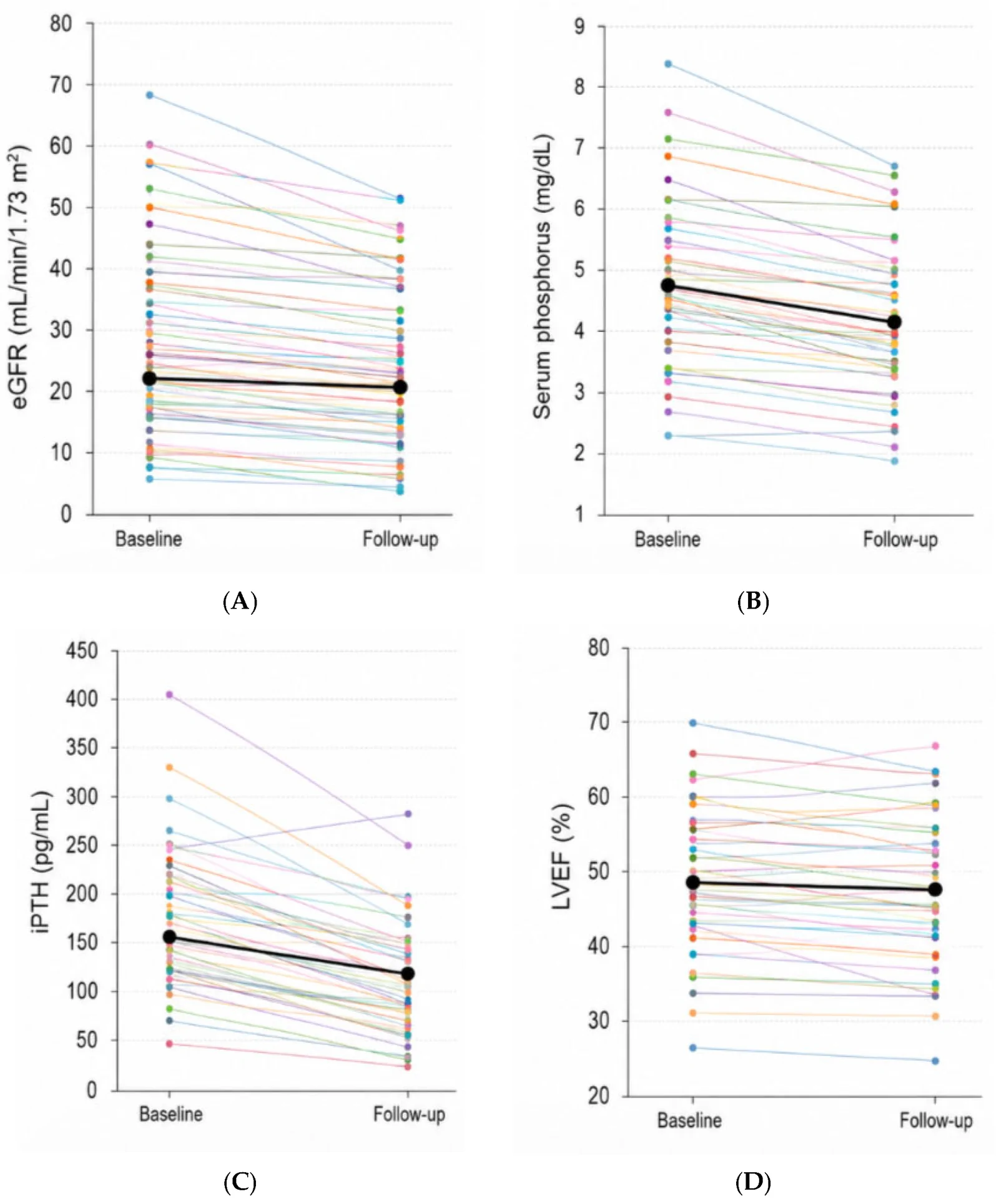
Individual paired renal, mineral–bone, and cardiovascular trajectories during follow-up. Paired baseline and follow-up values are shown for (**A**) estimated glomerular filtration rate (eGFR), (**B**) serum phosphorus, (**C**) intact parathyroid hormone (iPTH), and (**D**) left ventricular ejection fraction (LVEF). Each line connects measurements obtained from the same participant. The larger superimposed markers and connecting lines indicate the cohort-level median at baseline and follow-up. Individual trajectories illustrate between-patient variability and should be interpreted as longitudinal observations rather than effects attributable to the dietary intervention.

Median LVEF decreased from 48% to 47%, with an estimated paired change of −0.50 percentage points (95% CI, −1.00 to −0.50; *p* < 0.001). Although statistically significant, the absolute magnitude of this change was small. Systolic and DBP remained unchanged. NYHA class was stable in 61 patients (81.3%), improved in five (6.7%), and worsened in nine (12.0%). These NYHA transitions were summarized descriptively without formal inferential testing. Longitudinal changes are presented in Table 5, while selected individual trajectories for eGFR, serum phosphorus, iPTH, and LVEF are shown in Figure 3, illustrating the between-patient variability underlying the cohort-level findings.

### 3.8. Descriptive Findings According to Clinician-Recorded Adherence

Clinician-recorded adherence was categorized as high in 31 patients, intermediate in 30, and low in 14. Because adherence assessment was nonstandardized and follow-up duration differed substantially across categories, adherence-stratified outcome data are presented descriptively in Supplementary Table S3; no formal between-group inference was performed.

## 4. Discussion

### 4.1. Principal Findings

In this retrospective cohort of patients with advanced nondialysis CKD and chronic HF, body weight and BMI decreased during follow-up, and 29.3% of patients had a body-weight reduction of ≥5%, whereas serum albumin and total protein increased slightly. The body-weight reduction remained evident in the 181–366-day sensitivity analysis. Adherence-stratified outcomes were summarized descriptively and were not interpreted as evidence of an association between recorded adherence and subsequent nutritional changes, particularly because adherence assessment was nonstandardized and follow-up duration differed substantially across categories. eGFR decreased between baseline and final assessment, while several CKD–MBD biochemical variables changed during follow-up. Blood pressure showed little change, while NYHA functional class remained unchanged in 61 patients (81.3%), improved in five (6.7%), and worsened in nine (12.0%).

### 4.2. Interpretation of Nutritional Changes During the Dietary Protocol

The decline in body weight and BMI indicates that nutritional changes could not be assessed from serum proteins alone. Although albumin and total protein increased slightly at the cohort level, 29.3% of patients lost at least 5% of baseline weight, and 33.3% had documented signs suggestive of PEW. Albumin is influenced by inflammation, hepatic synthesis, protein losses, illness, and extracellular volume and may remain stable despite loss of muscle or fat mass [24]. The coexistence of preserved biochemical markers with reduced mid-arm muscle circumference, lower handgrip strength, appetite disturbance, and body-weight reduction in some patients supports a multidimensional approach to monitoring.

Validated multidimensional nutritional assessment tools, such as the Subjective Global Assessment, Malnutrition Inflammation Score, or GLIM criteria, were not applied systematically, and serial bioimpedance or other objective body-composition measurements were unavailable. Consequently, the present data cannot determine whether the observed anthropometric changes reflected loss of adipose tissue, muscle tissue, changes in fluid volume, or a combination of these factors. The chart-recorded appetite and muscle-related findings provide complementary clinical information but do not substitute for standardized nutritional assessment.

Body-weight reduction also persisted in the restricted analysis of patients followed for approximately 6–12 months, suggesting that the complete-cohort result was not explained solely by the patients with the longest observation periods. Nevertheless, the association between follow-up duration and cumulative percentage body-weight change indicates that observation time materially influenced the magnitude of the recorded reduction. The annualized estimate should also be interpreted cautiously because it assumes an approximately linear trajectory between two assessments.

Previous studies have generally reported preserved nutritional status during ketoanalogue-supplemented protein restriction. A meta-analysis of 16 randomized trials including 1344 patients with CKD stages 3–5 found no significant deterioration in albumin, lean body mass, mid-arm muscle circumference, or Subjective Global Assessment compared with low-protein diets alone [6]. Garneata et al. similarly observed stable anthropometric and biochemical indices during 15 months of a supplemented very-low-protein diet [25]. However, that trial included selected and motivated patients with verified adherence, adequate energy intake, and close dietary supervision. These conditions may not be reproduced consistently in routine clinical practice.

The margin of nutritional changes may be narrower in older patients and in those with advanced CKD, multimorbidity, impaired appetite, or reduced functional capacity. Findings from the EQUAL cohort suggested that prescribed protein restriction was not associated with greater nutritional decline or mortality overall, although outcomes were less favorable in patients older than 75 years and in those with poorer nutritional status or higher comorbidity burden [26]. Adequate energy provision therefore remains necessary, together with repeated assessment of weight trajectory, appetite, intake, muscle reserves, and physical function [24].

Interpretation of weight change is further complicated by chronic HF. Some reduction may have reflected changes in fluid status rather than loss of tissue mass, particularly in patients receiving loop diuretics. Weight alone is an imprecise marker of decongestion and correlates only modestly with urine output and symptom improvement in HF [27]. Thus, intentional or clinically favorable weight reduction related to improved volume control could not be reliably distinguished from body-weight reduction. Nevertheless, the accompanying appetite and muscle-related findings indicate that the observed body-weight reduction cannot be interpreted uniformly as either beneficial decongestion or nutritional deterioration. beneficial.

### 4.3. Dietary Adherence and Dietitian Monitoring

Adherence-stratified outcomes were reported descriptively because clinician-recorded adherence was not assessed using a standardized validated method and follow-up duration differed substantially across categories. Baseline albumin, total protein, HF phenotype, and NYHA class differed across the categories, and follow-up duration was strongly imbalanced. These differences limited the comparability of the adherence categories. Consequently, the numerical differences across adherence categories cannot be interpreted as evidence of an independent adherence effect on nutritional outcomes. Residual confounding and reverse causation remain plausible because declining appetite, intercurrent illness, functional deterioration, or emerging nutritional problems may themselves have reduced the ability to follow the prescribed regimen and may also have influenced the clinician-recorded adherence estimate.

Maintaining a ketoanalogue-supplemented very-low-protein diet is demanding. Restrictions on protein, phosphorus, sodium, and food choice may conflict with eating habits, family meals, social occasions, and weekend routines. Appetite limitation may also reduce the ability to meet energy targets in older patients with advanced CKD. Qualitative evidence across the CKD spectrum identifies taste preferences, cravings, cost, lack of time, dietary complexity, limited knowledge, and inadequate social support as barriers, while individualized guidance, food variety, alternative preparation methods, and family involvement act as facilitators [11]. Tablet burden is another obstacle because strict supplemented diets may require many ketoanalogue tablets daily [28].

Adherence measurement in the present study was nonstandardized. A numeric estimate was available for all patients, but the supporting method was documented in 30 through self-monitoring applications, food diaries, or dietary recall. For the remaining 45, the method was unspecified, and pill counts or biochemical validation were unavailable. This may have introduced recall and classification bias. Rizzetto et al. assessed adherence using two nonconsecutive food diaries, including one weekday and one weekend day, together with a 24 h recall at repeated dietitian visits, showing how repeated observations can capture variation over time [29].

Repeated counseling may be preferable to a single dietary instruction. Dietitian contact can identify appetite loss, social or weekend lapses, misunderstanding, and declining motivation early, allowing adjustment of meal plans, energy intake, and monitoring intensity. Adherence is therefore better considered a dynamic clinical characteristic that may vary during follow-up rather than a fixed patient characteristic.

### 4.4. Renal and CKD–MBD Changes

eGFR decreased between baseline and final follow-up, with a corresponding small increase in serum creatinine. Most patients remained in the same CKD G category, while serum urea did not change materially. Because renal function was assessed using only baseline and final measurements, these findings describe changes between two observations and do not establish an individual renal trajectory or rate of CKD progression. Biological and analytical variability in serum creatinine and eGFR may also have contributed to the observed differences.

Previous studies have associated ketoanalogue-supplemented protein restriction with slower loss of kidney function, although findings vary according to CKD severity, dietary exposure, adherence, and comparator treatment [30]. The present study had no untreated or comparison group, achieved dietary intake was not objectively measured, and concomitant treatments were incompletely characterized. The observed eGFR change therefore cannot be attributed to the prescribed dietary protocol or ketoanalogue supplementation and cannot be interpreted as evidence that CKD progression was slowed.

Phosphorus, the Ca × P, iPTH, and ALKP decreased, while serum calcium increased. These biochemical findings were observed during comprehensive clinical care and should not be attributed specifically to dietary protein restriction or ketoanalogue supplementation. In a randomized study of patients with CKD stages 4–5, lower protein intake reduced urinary urea nitrogen and phosphate excretion, although serum phosphorus and PTH did not differ between groups [31]. In the present cohort, achieved protein intake, catabolic state, hydration status, urinary urea nitrogen, and urea clearance were not measured. Accordingly, the unchanged serum urea concentration cannot be interpreted as evidence of reduced dietary nitrogen burden or successful metabolic control.

Interpretation of the CKD–MBD changes is additionally limited by the absence of standardized longitudinal information on concomitant CKD–MBD therapy. Phosphate binders, vitamin D compounds, calcimimetics, calcium supplementation, bicarbonate therapy, changes in kidney function, dietary prescription, and ketoanalogue supplementation may all have contributed, and their relative effects cannot be determined from the present data.

### 4.5. Cardiovascular Context of Nutritional and Renal Outcomes

All patients had chronic HF, most commonly HFmrEF, in the setting of advanced CKD. This cardiorenal profile complicates interpretation of body-weight change because venous congestion, edema, reduced appetite, intestinal edema, and diuretic-related fluid loss may affect both nutritional intake and measured weight. Some of the observed reductions may therefore have reflected decongestion, although the accompanying appetite and muscle-related findings support true nutritional deterioration in a subgroup.

Loop diuretics are frequently required to maintain euvolemia in patients with HF and CKD but may also contribute to volume depletion, hypotension, electrolyte disturbances, and transient renal-function changes [19,32,33]. Contemporary HF management also includes therapies that may influence renal function, blood pressure, volume status, and cardiovascular outcomes, including renin–angiotensin system blockade or angiotensin receptor–neprilysin inhibition, beta-blockers, mineralocorticoid receptor antagonists, and sodium–glucose cotransporter-2 inhibitors [19]. Longitudinal initiation, discontinuation, and dose changes of these therapies were not analyzed systematically in the present study. Likewise, changes in loop-diuretic dose, serial congestion severity, and dry weight were not captured consistently.

Provenzano et al. examined renal resistive index (RI) in a cross-sectional cohort of 73 patients with nondialysis CKD and found a mean RI of 0.67 ± 0.09. Higher RI values were independently associated with lower eGFR, previous cardiovascular disease, type 2 diabetes, smoking, and higher serum phosphorus. In the final multivariable model, eGFR, serum phosphorus, and cardiovascular disease accounted for the largest contributions to model fit (29.0%, 19.4%, and 12.2%, respectively). The authors suggested that elevated RI may reflect a more complex pattern of intrarenal damage extending beyond reduced kidney function alone. These findings reinforce the close interrelationship between renal dysfunction, mineral metabolism, and cardiovascular burden in patients with CKD and support interpreting renal and metabolic findings within a broader cardiorenal framework [20].

NT-proBNP concentrations are influenced by both cardiac stress and reduced kidney function but remain clinically informative in CKD [21]. Higher natriuretic peptide levels, advanced HF severity, renal dysfunction, anemia, and diuretic use have also been associated with poorer nutritional status and adverse outcomes in HF populations [22,23]. In the present cohort, however, NT-proBNP was available only at baseline and was not evaluated longitudinally; its role should therefore be interpreted as part of the underlying cardiovascular burden rather than as a marker of nutritional change during follow-up.

### 4.6. Clinical Implications

Clinical management of patients receiving a ketoanalogue-supplemented very-low-protein diet should begin with individualized protein and energy targets, adjusted for body size, appetite, comorbidity burden, and metabolic stability. Follow-up should include routine review of body weight, recent appetite, dietary tolerance, and adherence rather than relying on the initial prescription alone. Serum albumin may support assessment, but it should not be interpreted in isolation, particularly when inflammation, fluid overload, or HF may affect its value. A weight reduction of at least 5% should prompt early reassessment of energy intake, adherence, gastrointestinal symptoms, and treatment burden, together with closer dietitian review. Muscle status should also be examined through clinical inspection and, when available, mid-arm measurements, handgrip strength, or body-composition methods. In patients with HF, changes in weight must be interpreted alongside edema, congestion, blood pressure, and diuretic treatment to distinguish nutritional loss from fluid removal. Repeated counseling may be needed when adherence declines or the diet becomes difficult to sustain. Care is best coordinated across nephrology, nutrition, and cardiology, with treatment adjusted before nutritional deterioration becomes established.

### 4.7. Strengths and Limitations

This study has several strengths, including paired nutritional and renal assessments in a clinically complex cohort with advanced nondialysis CKD and chronic HF and simultaneous characterization of nutritional, renal, CKD–MBD, hematologic, and cardiovascular variables.

The main limitations are the retrospective single-center design, modest sample size, absence of a comparison group, and potential selection bias related to the requirement for paired baseline and final measurements. Dietary exposure was defined from prescription records; achieved protein and energy intake and actual ketoanalogue consumption were not objectively verified. Clinician-recorded adherence was assessed nonuniformly, with the supporting method undocumented in most patients, and the adherence groups differed in baseline characteristics and follow-up duration. These features limit interpretation of the descriptive adherence-stratified findings and preclude causal inference regarding adherence.

Follow-up varied from 181 to 730 days, and most longitudinal outcomes were based on only baseline and final measurements. Annualized body-weight and eGFR estimates therefore represent mathematical time-standardized approximations rather than measured trajectories. Standardized serial assessment of body composition, congestion, dry weight, and several concomitant CKD–MBD and HF treatments was unavailable, limiting interpretation of nutritional, renal, mineral, and cardiovascular changes. Events occurring outside the institution were not systematically captured, and the cohort cannot provide reliable estimates of dialysis initiation or mortality.

## 5. Conclusions

In this retrospective cohort of patients with advanced nondialysis CKD and chronic HF prescribed a ketoanalogue-supplemented very-low-protein diet, body weight and BMI decreased during follow-up, while serum albumin and total protein increased slightly. The prespecified primary outcome showed a reduction in body weight of −3.99% (Hodges–Lehmann estimate; 95% CI, −4.68 to −3.42), and a body-weight reduction ≥5% occurred in 29.3% of patients. Body-weight reduction also remained evident in the 181–366-day sensitivity analysis. eGFR decreased modestly between baseline and final assessment, while several CKD–MBD biochemical variables also changed. These observations support individualized nutritional monitoring but, in the absence of a comparison group and objectively verified dietary exposure, should not be interpreted as effects attributable to the prescribed dietary protocol.

## Figures and Tables

**Table 1:** Baseline clinical characteristics overall and according to clinician-recorded adherence category.

Variable	Overall Cohort	High Adherence	Intermediate Adherence	Low Adherence
	(*n* = 75)	(*n* = 31)	(*n* = 30)	(*n* = 14)
**Demographic and clinical characteristics**				
Age, years	68 (60–74)	65 (60–74)	70 (64–75)	72 (62–74)
Male sex, *n* (%)	45 (60.0)	16 (51.6)	19 (63.3)	10 (71.4)
Diabetes mellitus, *n* (%)	28 (37.3)	13 (41.9)	11 (36.7)	4 (28.6)
**Baseline CKD G category, ** ***n *** **(%)**				
G3b	14 (18.7)	5 (16.1)	8 (26.7)	1 (7.1)
G4	48 (64.0)	21 (67.7)	16 (53.3)	11 (78.6)
G5	13 (17.3)	5 (16.1)	6 (20.0)	2 (14.3)
**Nutritional characteristics**				
Weight, kg	75.85 ± 7.98	75.72 ± 8.66	75.31 ± 7.65	77.31 ± 7.44
BMI, kg/m^2^	26.9 ± 3.8	26.8 ± 3.6	26.9 ± 3.8	27.0 ± 4.6
Serum albumin, g/dL	3.72 (3.58–3.83)	3.83 (3.73–3.90)	3.71 (3.60–3.80)	3.39 (3.19–3.47)
Total protein, g/dL	6.80 (6.50–7.00)	6.90 (6.75–7.10)	6.75 (6.60–6.97)	6.20 (6.10–6.38)
**HF phenotype, ** ***n *** **(%)**				
HFpEF	28 (37.3)	15 (48.4)	9 (30.0)	4 (28.6)
HFmrEF	37 (49.3)	15 (48.4)	12 (40.0)	10 (71.4)
HFrEF	10 (13.3)	1 (3.2)	9 (30.0)	0 (0.0)
**NYHA class, ** ***n *** **(%)**				
II	55 (73.3)	28 (90.3)	18 (60.0)	9 (64.3)
III	19 (25.3)	3 (9.7)	11 (36.7)	5 (35.7)
IV	1 (1.3)	0 (0.0)	1 (3.3)	0 (0.0)
LVEF, %	48.0 ± 6.5	50.1 ± 5.4	46.0 ± 7.8	47.6 ± 4.1
NT-proBNP, pg/mL	3841 (2196–5388)	3167 (1917–4823)	4544 (3064–5893)	3543 (2016–4780)
AF, *n* (%)	21 (28.0)	9 (29.0)	10 (33.3)	2 (14.3)
CAD, *n* (%)	36 (48.0)	10 (32.3)	18 (60.0)	8 (57.1)

Data are presented as mean ± standard deviation, median [interquartile range], or number (percentage), as appropriate. High adherence was defined as ≥90%, intermediate adherence as 70–89%, and low adherence as <70%, based on the recorded numeric adherence percentage. Because adherence was not assessed using a standard, validated method and subgroup sizes were small and unequal, adherence-stratified baseline characteristics are presented descriptively without formal between-group hypothesis testing. The categories are retained to show the distribution of baseline characteristics and potential clinical imbalances and should not be interpreted as statistically distinct or validated adherence groups. No data were missing for the variables presented. Abbreviations: BMI, body mass index; CKD, chronic kidney disease; HFmrEF, heart failure with mildly reduced ejection fraction; HFpEF, heart failure with preserved ejection fraction; HFrEF, heart failure with reduced ejection fraction; LVEF, left ventricular ejection fraction; NT-proBNP, *N*-terminal pro-B-type natriuretic peptide; NYHA, New York Heart Association, AF, atrial fibrillation; CAD, Coronary artery disease.

**Table 2:** Prescribed dietary targets, ketoanalogue prescription and clinician-recorded adherence according to baseline CKD G category.

Variable	Overall Cohort	CKD G3b	CKD G4	CKD G5
	(*n* = 75)	(*n* = 14)	(*n* = 48)	(*n* = 13)
Prescribed protein target, g/kg/day	0.30 (0.30–0.35)	0.30 (0.30–0.30)	0.30 (0.30–0.35)	0.30 (0.30–0.40)
Prescribed energy target, kcal/kg/day	31 (29–33)	30 (28.5–31.8)	31 (28.8–32.0)	33 (30–34)
Prescribed ketoanalogue dose, tablets/day	15 (13.5–16)	16 (15–17)	15 (14–16)	13 (12–15)
Prescribed ketoanalogue dose, tablets/kg/day	0.198 (0.196–0.203)	0.196 (0.195–0.202)	0.199 (0.196–0.203]	0.198 (0.196–0.201)
Treatment duration, months	8 (7–12)	8 (7.3–9.8)	8 (7–12.3)	8 (7–10)
Adherence, %	88 (73.5–92.5)	88.5 (84.3–90.0)	87.5 (70.8–93.0)	88 (80–95)
**Adherence category, ** ***n *** **(%)**				
High adherence, ≥90%	31 (41.3)	5 (35.7)	21 (43.8)	5 (38.5)
Intermediate adherence, 70–89%	30 (40.0)	8 (57.1)	16 (33.3)	6 (46.2)
Low adherence, <70%	14 (18.7)	1 (7.1)	11 (22.9)	2 (15.4)

Data are presented as median [interquartile range] or number (percentage), as appropriate. Protein and energy values represent prescribed targets rather than systematically measured achieved intake. Clinician-recorded adherence was not assessed using a uniform validated method. Because the G3b and G5 subgroups were small and the purpose of this table was descriptive, no formal between-group hypothesis testing was performed. CKD G categories were retained because they represent established clinical categories and were used to describe the distribution of dietary prescription and recorded adherence across the cohort. Abbreviations: CKD, chronic kidney disease.

**Table 3:** Paired changes in anthropometric and biochemical changes during follow-up.

Variable	*n*	Baseline	Follow-Up	Individual Paired Change	Percentage or Annualized Change	Paired-Change Estimate (95% CI)	*p*-Value
Body weight, complete cohort, kg	75	75.85 ± 7.98	72.65 ± 7.09	−3.00 (−4.55 to −1.50)	−3.87% (−6.07 to −2.11)	−3.10 (−3.60 to −2.65)	<0.001
Percentage body-weight change, %	75	-	-	−3.87 (−6.07 to −2.11)	-	−3.99 (−4.68 to −3.42)	<0.001
BMI, kg/m^2^	75	26.9 ± 3.8	25.9 ± 3.9	−0.90 (−1.60 to −0.40)	−3.48% (−6.25 to −1.23)	−0.95 (−1.15 to −0.75)	<0.001
Albumin, g/dL	75	3.72 (3.58–3.84)	3.84 (3.57–3.96)	0.075 ± 0.117	1.92 ± 3.21%	0.075 (0.048 to 0.102)	<0.001
Total protein, g/dL	75	6.80 (6.50–7.00)	6.90 (6.50–7.10)	0.10 (0.00–0.10)	1.41% (0.00–1.49)	0.05 (0.05 to 0.10)	<0.001

Data are presented as mean ± standard deviation or median [interquartile range], as appropriate. Individual paired changes were calculated as follow-up minus baseline values, and percentage changes were calculated relative to each patient’s baseline value. Weight, BMI, and total protein were compared using the Wilcoxon signed-rank test; paired-change estimates represent Hodges–Lehmann estimates with bootstrap 95% confidence intervals. Albumin was compared using the paired-samples *t*-test, and its paired-change estimate represents the mean difference with a 95% confidence interval.

**Table 4:** Body-weight findings according to follow-up duration.

Outcome	181–366 Days (*n* = 62)	>366 Days (*n* = 13)
Baseline body weight, kg	75.57 ± 8.06	77.19 ± 7.75
Final body weight, kg	72.95 ± 7.20	71.22 ± 6.62
Mean body-weight change, kg	−2.62	−5.97
Body-weight reduction ≥5%, *n* (%)	10 (16.1)	12 (92.3)

Data are presented as mean ± standard deviation or number (percentage), as appropriate. Follow-up groups were defined according to the verified interval between baseline and final assessments. No formal between-group hypothesis testing was performed. Because only baseline and final body-weight measurements were consistently available, these findings represent observed cumulative changes rather than measured longitudinal trajectories and may also be influenced by changes in fluid status.

**Table 5:** Paired changes in renal, CKD–MBD, hematologic, and cardiovascular variables during follow-up.

Variable	Baseline	Follow-Up	Paired-Change Estimate	95% CI	*p*-Value	Effect Size
**Renal variables**						
eGFR, mL/min/1.73 m^2^	22.0 (16.0–26.5)	21.0 (14.5–25.0)	−1.50	−2.50 to −0.50	0.007	*r*_rb_ = −0.375
Annualized eGFR change, mL/min/1.73 m^2^/year	—	—	−1.96	−3.25 to −0.64	0.007	*r*_rb_ = −0.372
Creatinine, mg/dL	2.60 (2.15–3.43)	2.70 (2.15–3.60)	0.17	0.06 to 0.30	0.004	*r*_rb_ = 0.389
Urea, mg/dL	89.0 (77.0–127.5)	94.0 (76.0–114.0)	−1.50	−7.50 to 3.60	0.588	*r*_rb_ = −0.072
**Mineral–bone variables**						
Calcium, mg/dL	8.44 ± 0.77	8.85 ± 0.60	0.42	0.26 to 0.57	<0.001	*d*_z_ = 0.606
Phosphorus, mg/dL	4.70 (4.00–5.40)	4.20 (3.60–4.60)	−0.65	−0.85 to −0.45	<0.001	*r*_rb_ = −0.670
Ca × P, mg^2^/dL^2^	39.60 (34.43–44.02)	37.38 (32.27–40.37)	−3.17	−4.63 to −1.91	<0.001	*r*_rb_ = −0.552
iPTH, pg/mL	152.6 (122.2–197.3)	120.6 (83.3–161.0)	−44.03	−55.58 to −33.65	<0.001	*r*_rb_ = −0.828
ALKP, U/L	86.0 (69.5–111.0)	74.0 (58.5–89.5)	−17.0	−22.5 to −12.0	<0.001	*r*_rb_ = −0.732
**Hematologic variable**						
Hemoglobin, g/dL	10.81 ± 0.91	10.90 ± 1.03	0.08	−0.02 to 0.18	0.097	*d*_z_ = 0.194
**Cardiovascular variables**						
LVEF, %	48.0 (44.0–51.0)	47.0 (44.0–51.0)	−0.50	−1.00 to −0.50	<0.001	*r*_rb_ = −0.557
SBP, mmHg	103 (99–107)	102 (99–108)	0.00	−1.00 to 1.00	0.680	*r*_rb_ = 0.059
DBP, mmHg	63.0 (60.0–66.5)	63.0 (58.5–68.0)	0.00	−1.00 to 1.50	0.651	*r*_rb_ = −0.064

Data are presented as mean ± standard deviation or median [interquartile range], as appropriate. Paired-change estimates represent mean differences with 95% confidence intervals for normally distributed paired differences and Hodges–Lehmann estimates with bootstrap 95% confidence intervals for non-normally distributed variables. Effect sizes are reported as Cohen’s *d*_z_ or matched-pairs rank-biserial correlation *r*_rb_. Abbreviations: ALKP, alkaline phosphatase; Ca × P, calcium–phosphorus product; CI, confidence interval; CKD–MBD, chronic kidney disease–mineral and bone disorder; DBP, diastolic blood pressure; eGFR, estimated glomerular filtration rate; iPTH, intact parathyroid hormone; LVEF, left ventricular ejection fraction; SBP, systolic blood pressure.

## Data Availability

The data generated and analyzed during the current study are not publicly available because they contain sensitive clinical information and are subject to patient privacy and institutional ethical restrictions. De-identified data may be made available by the corresponding author upon reasonable request, subject to approval by the Ethics Committee of the Emergency University Hospital Bucharest and applicable institutional data-protection requirements.

## Supplementary Materials

The following supporting information can be downloaded at: https://www.mdpi.com/article/10.3390/nu18183069/s1, Table S1: Operational definitions, coding, units, and data sources. Table S2: Availability of data for variables included in longitudinal analyses. Table S3. Descriptive nutritional and renal findings according to clinician-recorded adherence category.

## Abbreviations

The following abbreviations are used in this manuscript:

AF	Atrial fibrillation
ALKP	Alkaline phosphatase
BMI	Body mass index
Ca × P	Calcium–phosphorus product
CKD	Chronic kidney disease
CKD–MBD	Chronic kidney disease–mineral and bone disorder
DBP	Diastolic blood pressure
eGFR	Estimated glomerular filtration rate
HF	Heart failure
HFmrEF	Heart failure with mildly reduced ejection fraction
HFpEF	Heart failure with preserved ejection fraction
HFrEF	Heart failure with reduced ejection fraction
iPTH	Intact parathyroid hormone
LVEF	Left ventricular ejection fraction
NT-proBNP	*N*-terminal pro-B-type natriuretic peptide
NYHA	New York Heart Association
PEW	Protein–energy wasting
SBP	Systolic blood pressure
