# Supplementary material for: Longitudinal Nutritional and Cardiorenal Changes Among Patients Prescribed a Ketoanalogue-Supplemented Very-Low-Protein Diet in Advanced Nondialysis Chronic Kidney Disease with Chronic Heart Failure: A Retrospective Cohort Study
Source: Nutrients. 2026 Sep 20;18(18):3069. doi: 10.3390/nu18183069 (PMC13610926; doi:10.3390/nu18183069)
Supplement: Supplementary file 1 [file nutrients-18-03069-s001.zip › Table_S1_Operational_definitions_coding_units_and_data_sources-2.pdf]

Table S1. Operational definitions, coding, units, and data sources

Variable	Operational definition	Unit/coding	Primary data source
**Demographic and assessment variables**			
Patient ID	Anonymized study-specific identifier assigned to each included patient and used for record linkage.	P001–P075	Compiled analytical database
Age	Age at the baseline assessment.	Years, continuous	Medical record
Sex	Sex recorded in the medical chart.	Male/Female	Medical record
Diabetes mellitus	Documented diagnosis of diabetes mellitus at baseline.	No/Yes	Medical history
Baseline assessment	First eligible assessment at initiation or confirmation of the ketoanalogue-supplemented dietary protocol.	Date	Medical and nutritional records
Final follow-up assessment	Last eligible assessment recorded no later than June 2026.	Date	Medical and nutritional records
Follow-up duration	Interval between the verified baseline and final assessment dates.	Days, continuous; years for time-standardized analyses	Derived from recorded dates
Restricted-duration sensitivity cohort	Patients with a verified follow-up interval of 181–366 days, used for the prespecified body-weight sensitivity analysis.	No/Yes	Derived variable
**Nutritional and anthropometric variables**			
Body weight	Body weight recorded at baseline and final follow-up.	kg	Nutritional/clinical record
Absolute body-weight change	Final body weight minus baseline body weight.	kg	Derived variable
Percentage body-weight change	Percentage change calculated as [(final weight − baseline weight)/baseline weight] × 100. Prespecified primary outcome.	%	Derived variable
Annualized percentage body-weight change	Percentage body-weight change divided by verified follow-up duration expressed in years. Used as a time-standardized sensitivity estimate rather than a measured longitudinal slope.	%/year	Derived variable
Body-weight reduction ≥5%	Reduction in body weight of at least 5% from baseline. Treated as an anthropometric outcome rather than a diagnostic criterion for malnutrition or PEW.	No/Yes	Derived variable
Body-weight reduction ≥10%	Reduction in body weight of at least 10% from baseline. Evaluated descriptively as an	No/Yes	Derived variable

	additional anthropometric outcome.		
Body mass index (BMI)	Body weight divided by height squared, assessed at baseline and final follow-up.	kg/m^2	Nutritional record/derived variable
BMI change	Final BMI minus baseline BMI; percentage change calculated relative to baseline when reported.	kg/m^2 and %	Derived variable
Serum albumin	Serum albumin concentration recorded at baseline and final follow-up.	g/dL	Laboratory record
Albumin change	Final serum albumin minus baseline serum albumin; percentage change calculated relative to baseline when reported.	g/dL and %	Derived variable
Total protein	Serum total protein concentration recorded at baseline and final follow-up.	g/dL	Laboratory record
Total-protein change	Final total protein minus baseline total protein; percentage change calculated relative to baseline when reported.	g/dL and %	Derived variable
Appetite-related findings	Qualitative appetite-related observations documented during clinical or dietitian follow-up, including reduced or fluctuating appetite or improvement when explicitly recorded.	Categorical, as documented	Dietitian and clinical records
Muscle-related findings	Routine-care muscle-related findings classified as absent, slight reduction in mid-arm muscle circumference without documented functional impairment, or reduced mid-arm muscle circumference accompanied by lower handgrip strength.	Categorical	Dietitian and clinical records
Chart-recorded signs suggestive of nutritional deterioration or PEW	Clinical findings documented in routine records as suggestive of nutritional deterioration or PEW. Complete standardized PEW diagnostic criteria were not available consistently; therefore, this variable was not considered confirmed PEW.	Absent/present; severity as documented	Dietitian and clinical records
**Dietary prescription, ketoanalogue exposure, and adherence**			

Prescribed protein target	Planned daily protein allowance expressed relative to actual body weight. Represents the prescription rather than measured achieved protein intake.	g/kg actual body weight/day	Dietitian/nutritional record
Prescribed energy target	Planned daily energy allowance expressed relative to actual body weight. Represents the prescription rather than measured achieved energy intake.	kcal/kg actual body weight/day	Dietitian/nutritional record
Ketoanalogue dose	Representative prescribed daily dose, calculated at approximately one tablet per 5 kg actual body weight and rounded to the nearest whole tablet.	Tablets/day	Medication and nutritional records
Weight-adjusted ketoanalogue dose	Prescribed daily ketoanalogue tablet dose divided by actual body weight.	Tablets/kg/day	Derived variable
Ketoanalogue treatment duration	Documented duration of the ketoanalogue-supplemented dietary protocol.	Months	Medication and nutritional records
Clinician-recorded adherence	Numeric routine-care estimate reflecting overall adherence to the prescribed protein-restricted diet, recommended energy target, and ketoanalogue use. The measure was not standardized or externally validated.	%, continuous	Dietitian/clinical record
Adherence category	Category derived from the clinician-recorded adherence percentage. Used only for descriptive supplementary summaries.	High, ≥90%; Intermediate, 70–89%; Low, <70%	Derived variable
Adherence-assessment method	Method explicitly documented for estimating adherence.	Self-monitoring application/Food diary/Dietary recall/Method not specified	Dietitian/clinical record
Enhanced dietitian intervention	Additional dietitian involvement documented in response to clinical or nutritional concerns.	No/Yes	Dietitian record
Intensified dietary counselling	Additional or intensified dietary counselling documented during follow-up.	No/Yes	Dietitian record
Oral nutritional supplementation considered	Documentation that oral nutritional supplementation was considered because oral intake was judged insufficient.	No/Yes	Dietitian/clinical record
**Renal variables**			

eGFR	Estimated glomerular filtration rate calculated from serum creatinine using the 2009 CKD-EPI creatinine equation and assessed at baseline and final follow-up.	mL/min/1.73 m^2	Laboratory record and derived variable
CKD G category	eGFR-based G category: G3a, 45–59; G3b, 30–44; G4, 15–29; G5, <15 mL/min/1.73 m^2. Baseline cohort included G3b, G4, and G5.	G3a/G3b/G4/G5	Derived from eGFR
CKD G-category transition	Comparison of final with baseline CKD G category. Used descriptively and based on the two available assessment points.	Less advanced/Stable/More advanced	Derived variable
Absolute eGFR change	Final eGFR minus baseline eGFR. Key secondary renal outcome.	mL/min/1.73 m^2	Derived variable
Annualized eGFR change	Absolute eGFR change divided by verified follow-up duration expressed in years. Used as a time-standardized sensitivity estimate rather than an individual renal slope.	mL/min/1.73 m^2/year	Derived variable
Serum creatinine	Serum creatinine concentration recorded at baseline and final follow-up.	mg/dL	Laboratory record
Serum urea	Serum urea concentration recorded at baseline and final follow-up.	mg/dL	Laboratory record
**CKD–MBD variables**			
Serum calcium	Serum calcium concentration recorded at baseline and final follow-up.	mg/dL	Laboratory record
Serum phosphorus	Serum phosphorus concentration recorded at baseline and final follow-up.	mg/dL	Laboratory record
Calcium–phosphorus product (Ca × P)	Product of serum calcium and serum phosphorus concentrations.	mg^2/dL^2	Derived variable
Intact parathyroid hormone (iPTH)	Serum intact parathyroid hormone concentration recorded at baseline and final follow-up.	pg/mL	Laboratory record
Alkaline phosphatase (ALKP)	Serum alkaline phosphatase concentration recorded at baseline and final follow-up.	U/L	Laboratory record
**Hematologic variables**			
Hemoglobin	Hemoglobin concentration recorded at baseline and final follow-up.	g/dL	Laboratory record

Anemia grade	Severity category documented or assigned from the available hematologic information at baseline.	Categorical	Laboratory/clinical record
Iron deficiency	Documented iron deficiency in the available clinical or laboratory record.	No/Yes	Laboratory/clinical record
**Cardiovascular variables**			
Chronic heart failure	Established chronic HF documented in all patients in the final linked cohort. HF was a cohort-defining characteristic of the linked dataset rather than a separate eligibility criterion.	Yes for all included patients	Medical history
HF phenotype	Classification based on LVEF: HFrEF, LVEF ≤40%; HFmrEF, LVEF 41–49%; HFpEF, LVEF ≥50%.	HFrEF/HFmrEF/HFpEF	Echocardiography
NYHA functional class	New York Heart Association functional class recorded at baseline and final follow-up as a symptom-based measure of functional limitation.	II/III/IV	Medical record
NYHA functional-class transition	Comparison of final NYHA class with baseline class. Summarized descriptively without formal inferential testing.	Improved/Stable/Worsened	Derived variable
LVEF	Left ventricular ejection fraction recorded at baseline and final follow-up.	%	Echocardiography
NT-proBNP	N-terminal pro-B-type natriuretic peptide concentration. Serial measurements were not systematically available; therefore, NT-proBNP was analyzed as a baseline cardiovascular marker only.	pg/mL	Laboratory record
Atrial fibrillation (AF)	Documented diagnosis of atrial fibrillation in the available medical record.	No/Yes	Medical history
Coronary artery disease (CAD)	Documented diagnosis of coronary artery disease in the available medical record.	No/Yes	Medical history
Systolic blood pressure (SBP)	Systolic blood pressure recorded at baseline and final follow-up.	mmHg	Medical record
Diastolic blood pressure (DBP)	Diastolic blood pressure recorded at baseline and final follow-up.	mmHg	Medical record
Loop-diuretic treatment	Documented loop-diuretic treatment. Longitudinal dose	No/Yes	Medication record

changes were not captured consistently.

Note: Unless otherwise specified, longitudinal variables refer to measurements obtained at the baseline and final follow-up assessments. No predefined maximum time window was imposed for laboratory or echocardiographic measurements relative to these assessment dates. When measurements occurred on different dates, the value closest to the corresponding assessment date was retained. Missing values were not imputed. Variables retained in paired longitudinal analyses had both baseline and follow-up values available; variable-specific availability is reported in Supplementary Table S2. Annualized body-weight and eGFR changes are mathematical time-standardized sensitivity estimates based on two assessment points and should not be interpreted as measured longitudinal slopes. Clinician-recorded adherence was nonstandardized and was used only for descriptive supplementary summaries. Body-weight reduction and chart-recorded nutritional findings were not considered diagnostic of malnutrition or confirmed PEW. CKD G-category classification was based on eGFR and does not represent complete KDIGO CGA classification because albuminuria was unavailable.

Abbreviations: AF, atrial fibrillation; ALKP, alkaline phosphatase; BMI, body mass index; CAD, coronary artery disease; Ca × P, calcium–phosphorus product; CKD, chronic kidney disease; CKD–MBD, chronic kidney disease–mineral and bone disorder; DBP, diastolic blood pressure; eGFR, estimated glomerular filtration rate; HF, heart failure; HFmrEF, heart failure with mildly reduced ejection fraction; HFpEF, heart failure with preserved ejection fraction; HFrEF, heart failure with reduced ejection fraction; iPTH, intact parathyroid hormone; LVEF, left ventricular ejection fraction; NT-proBNP, N-terminal pro-B-type natriuretic peptide; NYHA, New York Heart Association; PEW, protein–energy wasting; SBP, systolic blood pressure.
