# Supplementary material for: Longitudinal Nutritional and Cardiorenal Changes Among Patients Prescribed a Ketoanalogue-Supplemented Very-Low-Protein Diet in Advanced Nondialysis Chronic Kidney Disease with Chronic Heart Failure: A Retrospective Cohort Study
Source: Nutrients. 2026 Sep 20;18(18):3069. doi: 10.3390/nu18183069 (PMC13610926; doi:10.3390/nu18183069)
Supplement: Supplementary file 1 [file nutrients-18-03069-s001.zip › Table_S2_Availability_of_data_for_variables_included_in_longitudinal_analyses-1.pdf]

Table S2. Availability of data for variables included in longitudinal analyses

Domain	Variable	Baseline, n	Follow-up, n	Paired/derived analysis, n
Anthropometric	Body weight	75	75	75
	BMI	75	75	75
Biochemical nutritional	Serum albumin	75	75	75
	Total protein	75	75	75
Renal	eGFR	75	75	75
	CKD G category	75	75	75
	Serum creatinine	75	75	75
	Serum urea	75	75	75
CKD–MBD	Serum calcium	75	75	75
	Serum phosphorus	75	75	75
	Calcium–phosphorus product	75	75	75
	iPTH	75	75	75
	Alkaline phosphatase	75	75	75
Hematologic	Hemoglobin	75	75	75
Cardiovascular	LVEF	75	75	75
	NYHA functional class	75	75	75
	Systolic blood pressure	75	75	75
	Diastolic blood pressure	75	75	75
Derived longitudinal outcomes	Percentage body-weight change	—	—	75
	Annualized percentage body-weight change	—	—	75
	Body-weight reduction ≥5%	—	—	75
	Body-weight reduction ≥10%	—	—	75
	Annualized eGFR estimate	—	—	75
	CKD G-category transition	—	—	75
	NYHA functional-class transition	—	—	75

Baseline and follow-up counts indicate the number of patients with an available value at the corresponding assessment. Paired-analysis counts indicate patients with both baseline and follow-up values or, for derived outcomes, the number for whom the outcome could be calculated. Missing values were not imputed. All variables retained in the paired longitudinal analyses had both baseline and follow-up values available for all 75 patients. The body-weight sensitivity analysis restricted to 181–366 days included 62 patients by design, while 13 patients had follow-up >366 days; these subgroup sizes reflect the follow-up-duration categories used for the sensitivity and descriptive duration-stratified analyses rather than missing data. NT-proBNP was treated as a baseline marker because serial measurements were not consistently available and was therefore not included among the longitudinal variables. Chart-recorded appetite and muscle-related findings were obtained at nonstandardized times and were analyzed descriptively rather than as paired longitudinal measurements.
