# Supplementary material for: Longitudinal Nutritional and Cardiorenal Changes Among Patients Prescribed a Ketoanalogue-Supplemented Very-Low-Protein Diet in Advanced Nondialysis Chronic Kidney Disease with Chronic Heart Failure: A Retrospective Cohort Study
Source: Nutrients. 2026 Sep 20;18(18):3069. doi: 10.3390/nu18183069 (PMC13610926; doi:10.3390/nu18183069)
Supplement: Supplementary file 1 [file nutrients-18-03069-s001.zip › Table_S3_Descriptive_nutritional_and_renal_findings_according_to_clinician_recorded_adherence_category.pdf]

Table S3. Descriptive nutritional and renal findings according to clinician-recorded adherence category.

Outcome	High recorded adherence (*n* = 31)	Intermediate recorded adherence (*n* = 30)	Low recorded adherence (*n* = 14)
Body-weight change, %	−1.84	−3.93	−8.04
Body-weight reduction ≥5%, *n* (%)	0 (0.0)	9 (30.0)	13 (92.9)
Follow-up >12 months, *n* (%)	0 (0.0)	0 (0.0)	13 (92.9)
Albumin change, g/dL	0.12 [0.08–0.20]	0.08 [0.01–0.16]	−0.08 [−0.16 to −0.02]
Chart-recorded signs suggestive of nutritional deterioration, *n* (%)	0 (0.0)	11 (36.7)	14 (100)
Annualized eGFR change, mL/min/1.73 m^2/year	−1.33 [−3.24 to 1.86]	−2.39 [−7.88 to 1.69]	−3.21 [−5.79 to −1.38]
Phosphorus change, mg/dL	−0.50 [−0.70 to −0.20]	−0.85 [−1.20 to −0.25]	−0.70 [−1.48 to −0.50]
iPTH change, pg/mL	−26.20 [−59.95 to −11.25]	−46.50 [−84.35 to −22.33]	−42.15 [−77.33 to −33.03]

Note: Continuous data are presented as median [interquartile range], except for percentage body-weight change, for which only the group median is shown; categorical data are presented as number (percentage). Adherence categories were based on the clinician-recorded numeric adherence estimate: high, ≥90%; intermediate, 70–89%; and low, <70%. Adherence was not assessed using a standardized validated method, and its timing was not standardized. Follow-up duration also differed substantially across categories, with all patients followed for >12 months belonging to the low recorded-adherence group. Accordingly, these data are presented descriptively only; no formal between-group hypothesis testing was performed, and the observed numerical differences should not be interpreted as evidence of an independent or causal adherence effect.

Abbreviations: eGFR, estimated glomerular filtration rate; iPTH, intact parathyroid hormone.
